# Response to Buonocore et al. Comments on Wendt Hess et al. “Assessing Agreement in Exposure Classification between Proximity-Based Metrics and Air Monitoring Data in Epidemiology Studies of Unconventional Resource Development.” *Int. J. Environ. Res. Public Health* 2019, *16*, 3055

**DOI:** 10.3390/ijerph17020512

**Published:** 2020-01-14

**Authors:** Judy Wendt Hess, Gerald Bachler, Fayaz Momin, Krystal Sexton

**Affiliations:** 1 Shell Health Risk Science Team, Shell Oil Company, 150 North Dairy Ashford, Houston, TX 77079, USA; fayaz.momin@shell.com (F.M.); krystal.sexton@shell.com (K.S.); 2 Shell Health Risk Science Team, Shell International B.V., Carel Van Bylandtlaan 16, 2596 HR The Hague, The Netherlands; g.bachler@hotmail.com

We appreciate the comments by Buonocore et al., and the opportunity to respond. We acknowledged in our paper [1] that distance proxies could potentially reflect a number of exposure pathways. However, this does not diminish the value of examining air, which is often the focus of policy formulation and regulation.

Our analysis did not assess whether continuous values of well activity (WA) and air pollutant concentrations were correlated. The question we asked was whether there was general agreement between exposure classifications based on WA and air pollutant concentrations, which, at the end of the day, is how potential exposure misclassification can be assessed. *WA models do not represent theoretical exposures related to well density.* For the air pathway, calculated WA values are proxies for pollutants assumed to be emitted from well sites, which travel and persist in the air in sufficient concentrations that, upon reaching Pennsylvania residents, could potentially initiate the development of disease. If WA metrics worked as envisioned, ‘high’ exposure—as a consequence of living closer to more and larger wells–would be evidenced by relatively high concentrations of the pollutants assumed to be emitted from the wells. ‘Very low’ exposure would be evidenced by relatively low concentrations of air pollutants assumed to be emitted from the wells. This was what we tested in our analysis, which included benzene and almost all of the U.S. Environmental Protection Agency’s criteria pollutants.

We suspect that the WA approach was developed to leverage the rich health outcomes data available from the Geisinger Health System. However, the challenge these researchers faced was that population centers within the Geisinger catchment area are not, for the most part, located in areas of Pennsylvania with significant shale development. Neither are the Geisinger medical centers from which (e.g., asthma) cases were drawn. In other words, most of the study population included in the Geisinger studies did not live anywhere near shale development. Figure 1 and Figure 2 below, adapted from Casey et al. [2] and Rasmussen et al. [3], illustrate this.

Why does this matter? Buonocore et al. addressed this in their comments:

- The pollutants included in our analysis “have fairly short atmospheric lifetimes and can only travel short distances.” As a result, only a small proportion of subjects included in these studies could reasonably be expected to have any exposure to these pollutants from shale development operations via the air pathway.
- Potential exposure from oil and gas (O&G) operations “vary both spatially and temporally, and act over short distances and time scales.” WA models assume that all wells, in the same phase of development (the durations of which were predetermined, not based on actual well data), continuously emit pollutants in all directions. There is no consideration of varying meteorological conditions mentioned by Buonocore et al. (e.g., wind direction, cloud cover, wind speed). None of these are realistic assumptions.
- The pollutants included in our analysis are not unique to shale development, but rather “come from a number of sources common in the area including vehicular traffic, combustion of coal and gas for electricity generation and home heating, not to mention natural gas processing and existing conventional O&G wells.” WA models do not take any of these other point and mobile sources into account. This is especially significant given that the models estimate exposure for study subjects who are, for the most part, far from any unconventional development.

Based on numbers provided in Casey et al. [2], we estimate that fewer than 10% of the 10,496 births included in the study were in areas potentially exposed to emissions from unconventional development activity. Including the other 90% in the study, particularly when the data are divided into quartiles, not only dilutes the population at risk, but also results in exposure categories with little meaning. Figures 7–10 in our paper show what are essentially random exposure assignments across the four categories, a pattern confirmed by our weighted kappa analysis.

We believe that the points raised by Buonocore et al. actually argue against the validity of WA models for assigning exposure in epidemiology studies, not against the validity of our analysis that evaluated them. These authors list many requirements for proper exposure estimation, yet do not acknowledge the potential for significant exposure misclassification in the Geisinger studies, which included none of these elements. We agree that epidemiology studies have a vital role to play in informing public policy around O&G development. However, if the Geisinger studies suffer from such a fundamental source of bias, their contribution to the assessment of health risks is limited. This is why, in our paper, we urged caution when relying on these studies for risk communication or policy development.

## Figures and Tables

**Figure 1:**
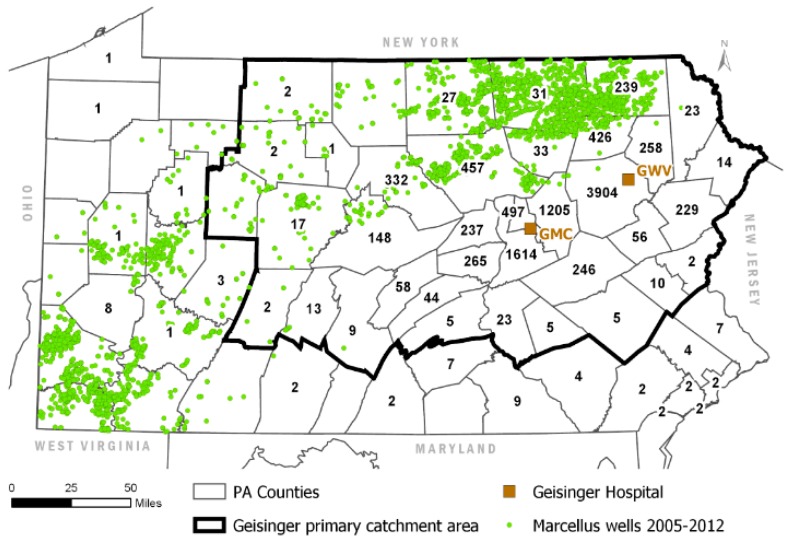
Adapted from Casey et al. [2], showing the Geisinger primary catchment area relative to the location of unconventional wells spudded or in production during the period 2005–2012. The numbers within the county boundaries describe the distribution of the study population (*n* = 10,496) across the state and indicate the number of babies born to mothers living in each county. GMC = Geisinger Medical Center, GWV = Geisinger Wyoming Valley. Figure was regenerated with data available through Shell’s licensing agreements with Esri and IHS Markit Ltd.

**Figure 2:**
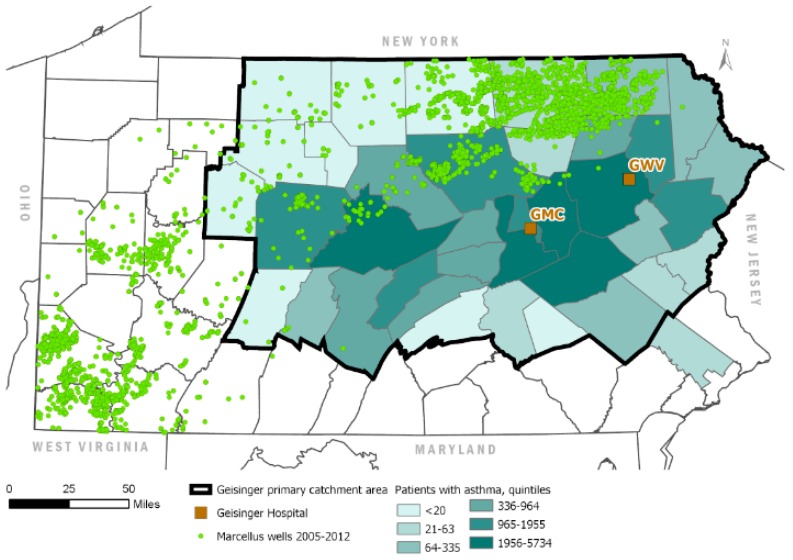
Adapted from Rasmussen et al. [3], showing the Geisinger primary catchment area relative to the location of unconventional wells spudded or in production during the period 2005–2012. Shading indicates the distribution of Geisinger asthma patients comprising the study population by county. GMC = Geisinger Medical Center, GWV = Geisinger Wyoming Valley. Figure was regenerated with data available through Shell’s licensing agreements with Esri and IHS Markit Ltd.
